# Experimental verification of subthreshold laser therapy using conventional pattern scan laser

**DOI:** 10.1371/journal.pone.0184392

**Published:** 2017-09-07

**Authors:** Tomoyasu Shiraya, Satoshi Kato, Fumiyuki Araki, Takashi Ueta, Hitoshi Abe, Nobuharu Asai

**Affiliations:** 1 Department of Ophthalmology, Graduate School of Medicine, Tokyo, Japan; 2 NIDEK Co., Ltd., Gamagori, Japan; University of Florida, UNITED STATES

## Abstract

**Purpose:**

Leading-edge therapeutic laser technologies are not available at every medical facility; therefore, alternative approaches incorporating novel advances in digital and laser technology into more readily available conventional methods have generated significant research interest. Using a rabbit model, this study investigated whether the algorithm used in the Endpoint Management (EM) software system of the latest devices could enable subthreshold laser treatment in conventional retinal tissue laser therapy systems.

**Materials and methods:**

Two types of devices were used, the PASCAL Streamline 577 and the MC 500-Vixi™, and the laser method was classified into three categories: EM; single-shot using PASCAL with arbitrary energy settings (PSS-SDM); and MC500-Vixi^TM^ (VX-SDM), which were performed in eight eyes from four Dutch-Belted rabbits. In EM, 100 mW (100%) was set as a landmark, and the laser energy parameters were gradually decreased to 80%, 60%, 50%, 40%, 30%, 20%, and 10%, using a 2 × 3 square pattern. In PSS-SDM and VX-SDM, as control, the laser energy was gradually decreased to 100, 80, 60, 50, 40, 30, 20, and 10 mW. The laser settings were fixed at 200 μm, 20 ms, and a wavelength of 577 μm. To identify and compare the extent of tissue damage at each spot size, optical coherence tomography (OCT) and histological findings were used to construct a three-dimensional histopathology image using a confocal laser scanning fluorescence microscope.

**Results:**

The spot size at 50% setting on EM was 7183 μm^2^; PSS-SDM required 50 mW (5503 μm^2^) to 60 mW (10279 μm^2^) and VX-SDM required 50 mW (7423 μm^2^) to create the approximate spot size. Furthermore, at 50 mW of PSS-SDM and VX-SDM, the extent of tissue damage in all three methods was generally in accord with the outer nuclear layer by OCT and inner nuclear layer by histopathological imaging.

**Conclusion:**

These findings suggest that it may be possible to perform subthreshold laser therapy using approximations from the EM algorithm.

## Introduction

Diabetic macular edema (DME) is estimated to affect 21 million individuals worldwide. Furthermore, DME affects approximately 29% of diabetic patients with a disease duration ≥20 years and remains one of the most frequent causes of vision loss in this patient group [1]. Retinal laser photocoagulation for DME and, especially, diffuse macular edema, has been investigated. Although the Early Treatment of Diabetic Retinopathy Study (ETDRS) reported on the effectiveness of macular grid laser photocoagulation for diffuse DME and concluded that it reduced vision loss by approximately 50% at three years’ follow-up [2], subsequent reports have described complications, such as ring scotoma and central vision loss, due to the expansion and fusion of coagulation spots during scleral changes [3]. Later, technical improvements were incorporated into the original ETDRS photocoagulation method, and the Diabetic Retinopathy Clinical Research Network (DRCR.net) reported it as a modified ETDRS focal/grid photocoagulation protocol [4]; nevertheless, the risk for adverse events remain. Based on this background, along with advances in retinal photocoagulator technology, a review of treatment settings is ongoing.

PASCAL^®^ (OptiMedica Corp, Santa Barbara, California), a new photocoagulator system introduced in 2006, uses a high-power, short-duration (10 msec to 30 msec) method, and is also equipped with a pattern scanning laser [5], which affords the advantage of less tissue damage to the inner retina [6]. Furthermore, previous studies have found that a high-power, short-duration method leads to minimal expansion of coagulation spots compared with conventional methods [7]. More recently, subthreshold laser treatment (STLT) for the treatment of macular edema has attracted attention. This method targets the retinal pigment epithelium (RPE), and minimally affects the outer retina or choroid, with no apparent visible color changes in treated areas [8,9]. The STLT method includes an 810 nm micropulse diode laser that continuously delivers extremely short-duration laser irradiation. The above-mentioned PASCAL system also has an STLT-capable model equipped with the Endpoint Management (EM) software package, which enables STLT using computational control of pulse energy to achieve the desired tissue effects. Because STLT does not cause retinal photoreceptor damage or cell death, which can lead to more serious problems, postoperative scotoma has not been reported, with significantly fewer potential complications compared with conventional macular grid photocoagulation. In contrast to conventional methods, which sometimes result in damage to the RPE, the rationale for STLT is based on the assumption that performing laser application at an extremely low power at which laser spots are invisible stimulates the healing response of the RPE to thermal injury by activating a cellular cascade [10]. Considering the invasiveness of conventional retinal tissue laser therapy, excessive laser energies lead to problems such as retinal bleeding and inflammation. Conversely, if the laser energy is too low, no therapeutic effect can be achieved. Although the EM program maximizes the therapeutic effect using a specific algorithm [10], the optimal energy settings (i.e., %) remain unclear and are an ongoing topic of research.

Although EM is believed to be useful for treating DME, it is not available at every medical facility. However, if the laser application characteristics of the EM algorithm can be optimized, there is a possibility that they can be effectively applied using a conventional photocoagulator not equipped with the EM software. In this study, experimental retinal photocoagulation was performed in Dutch-Belted rabbits using EM and a conventional photocoagulator to investigate the possibility of achieving approximate laser spots based on EM according to histopathology and optical coherence tomography (OCT) findings.

## Materials and methods

### 2.1. Experimental protocols

Retinal laser treatment was performed with two types of laser using EM and a conventional method as control. Immunostaining was then performed on retinal tissue sections that included each coagulated spot lesion. To determine the energy levels that corresponded with spot sizes between EM and control, the area of each spot was measured from histological methods. To determine the energy levels that corresponded with identifiable tissue damage in the lesions between EM and control, three-dimensional computerized histopathology images were re-constructed from the retinal tissue and OCT findings. From these results, the energy settings to create lesions approximating EM using the conventional model were comprehensively evaluated.

### 2.2. Photocoagulation system and laser protocol

Two types of devices were used: the PASCAL Streamline 577 ™ (Topcon Medical Laser Systems, Inc., Santa Clara, CA, USA); and the MC 500-Vixi ™ (NIDEK, Gamagori, Japan). The laser method was classified into 3 categories: EM; single-shot using PASCAL with arbitrary energy settings (PASCAL short-duration method [PSS-SDM]); and MC500-Vixi^TM^ (VX short-duration method [VX-SDM]). In EM, 100 mW (100%) was set as a landmark (i.e., marker spot), and the laser energy parameters were gradually decreased to 80%, 60%, 50%, 40%, 30%, 20%, and 10%, using a 2 × 3 square pattern. Regarding the 2 × 3 pattern, four landmark spots of the same energy and two EM-adjusted spots were created. One landmark spot and all spots from EM-adjusted spots at each energy setting were examined.

In PSS-SDM and VX-SDM, as control, the laser energy was gradually decreased to 100, 80, 60, 50, 40, 30, 20, and 10 mW. An experimental MC 500-Vixi™ device that enabled minimum energy settings up to 30 mW was used to align with that of the PASCAL system. The laser settings were fixed at 200 μm (spot size), 20 ms, and a wavelength of 577 μm.

### 2.3. Laser application and pathology of retinal tissue

Eight eyes from four Dutch-Belted rabbits (23 weeks of age, 2.5 kg) were used according to and with approval from the Institutional Animal Care and Use Committee of the University of Tokyo (Tokyo, Japan [approval number M-P15-128]). All experiments were performed under anesthesia, and all efforts were made to minimize suffering. Pupil dilation was achieved using one drop each of 1% tropicamide and 2.5% phenylephrine hydrochloride. Topical tetracaine hydrochloride 0.5% was used for local anesthesia. The rabbits were sedated by intramuscular injection using 3 mL of a ketalar:celactar (8:2) mixture. Wide-field retinal contact lens (Trans Equator^®^; Volk Optical, Mentor, OH, USA) was used with hydroxypropyl methylcellulose as a contact gel.

**Fig 1:**
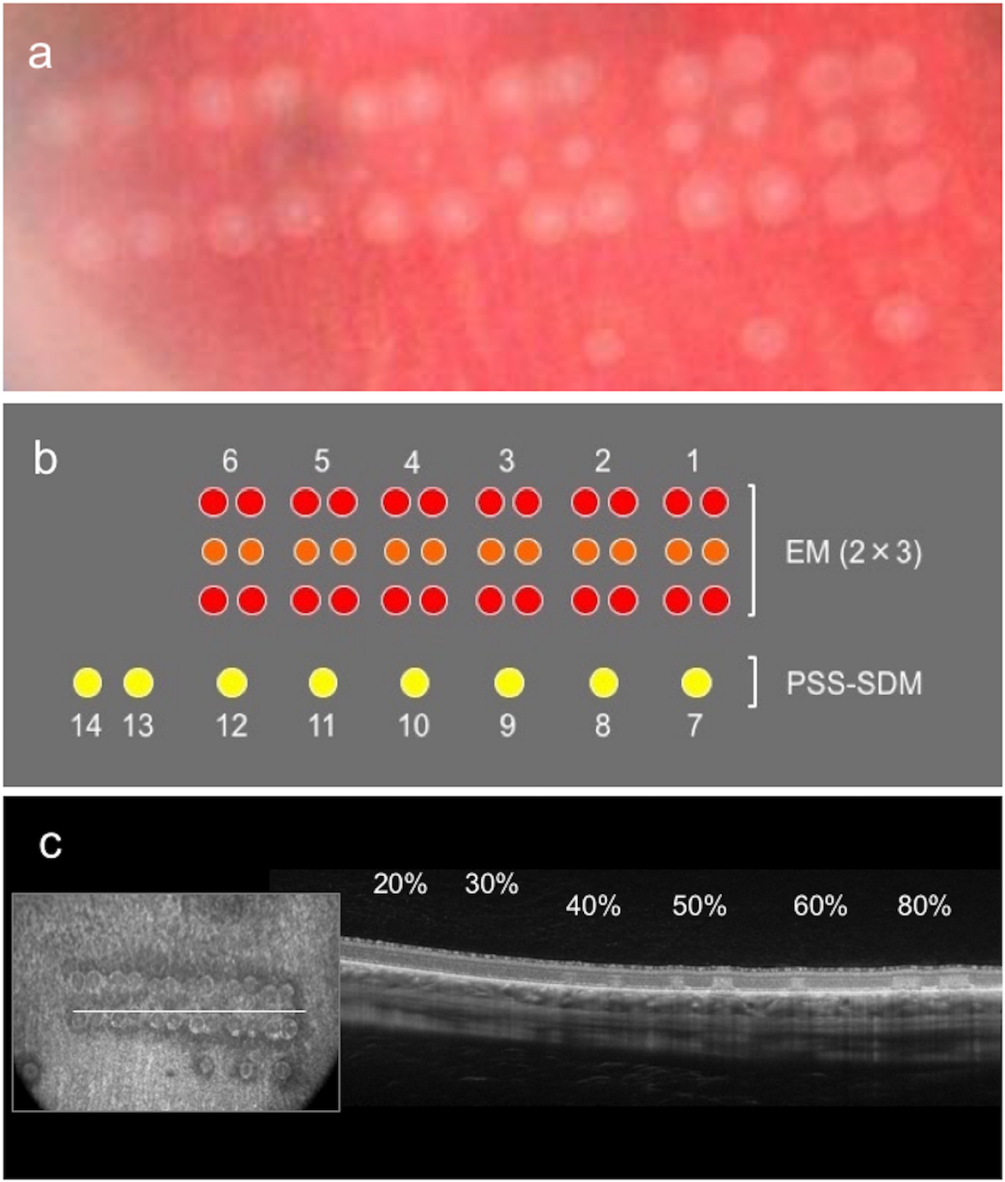
Schematic representation of laser pattern and the extent of tissue damage according to optical coherence tomography. (a) Color fundus photograph of laser application using PASCAL (Streamline 577™ (Topcon Medical Laser Systems, Inc., Santa Clara, CA, USA) with Endpoint Management (EM) using a 2×3 square pattern and single shot using the PASCAL short-duration method (PSS-SDM) imaged immediately after laser treatment. (b) Schematic representation of the laser pattern in the retina. The laser application settings were fixed at 200 μm (spot size), 20 ms, and a wavelength of 577 μm. Numbers 1 to 6 represent laser spots using EM (2×3 square pattern), while 7 to 14 represent laser spots created by PSS-SDM. (c) Optical coherence tomography immediately after laser application was performed with slices containing laser spots, and the extent of tissue damage at the spot lesion were identified. Percentages indicate the energy setting at each lesion.

Laser treatment was performed by a single surgeon (K.S.) at a specified area that was inferior to the optic disc under fixed conditions using the PASCAL^®^ and MC 500-Vixi™ devices equipped with original adapters for rabbits. Color fundus photographs (AFC-330; NIDEK, Gamagori, Japan; and OCT [RS-3000 advance]; NIDEK, Gamagori, Japan) including each laser spot were taken immediately after treatment (Fig 1).

**Fig 2:**
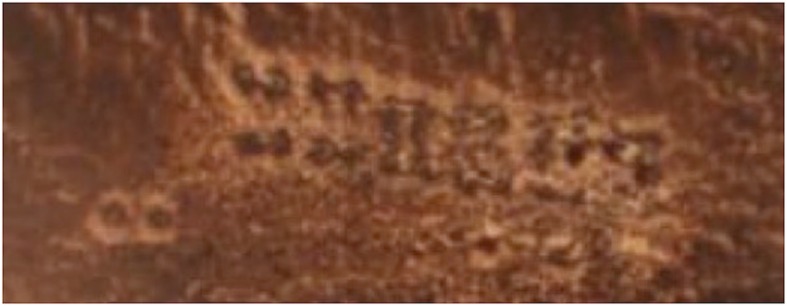
Preparation of retinal extension sections. Retinal extension sections (2 cm^2^; inferior to the optic disc) fixed in 4% paraformaldehyde including the laser spot.

After experimental laser application, the animals were housed for 48 h. After intramuscular injection of a 4 mL ketalar:serracel (8:2) mixture for euthanasia and exsanguination by carotid artery amputation, the eyes were enucleated and fixed in 4% paraformaldehyde in phosphate-buffered saline (PBS) overnight. The retina was separated from the eye-cap and embedded in 0.5% Triton X-100 in PBS for 10 min and frozen. Retinal tissue section specimens (2 cm^2^; inferior to the optic disc) including the irradiation pattern were prepared (Fig 2). The slides were dried for 30 min at room temperature, washed with 0.5% Triton X-100 in PBS, and then incubated with primary antibody (mouse anti-rhodopsin, 4D2, Millipore, USA) and DAPI stain at room temperature. Slides were subsequently washed with PBS three times for 5 min each, and subsequently incubated with secondary antibody (Alexa 488-conjugated goat anti-mouse IgG, Molecular Probes, USA) at room temperature.

### 2.4. Estimation of photocoagulation spot size

**Fig 3:**
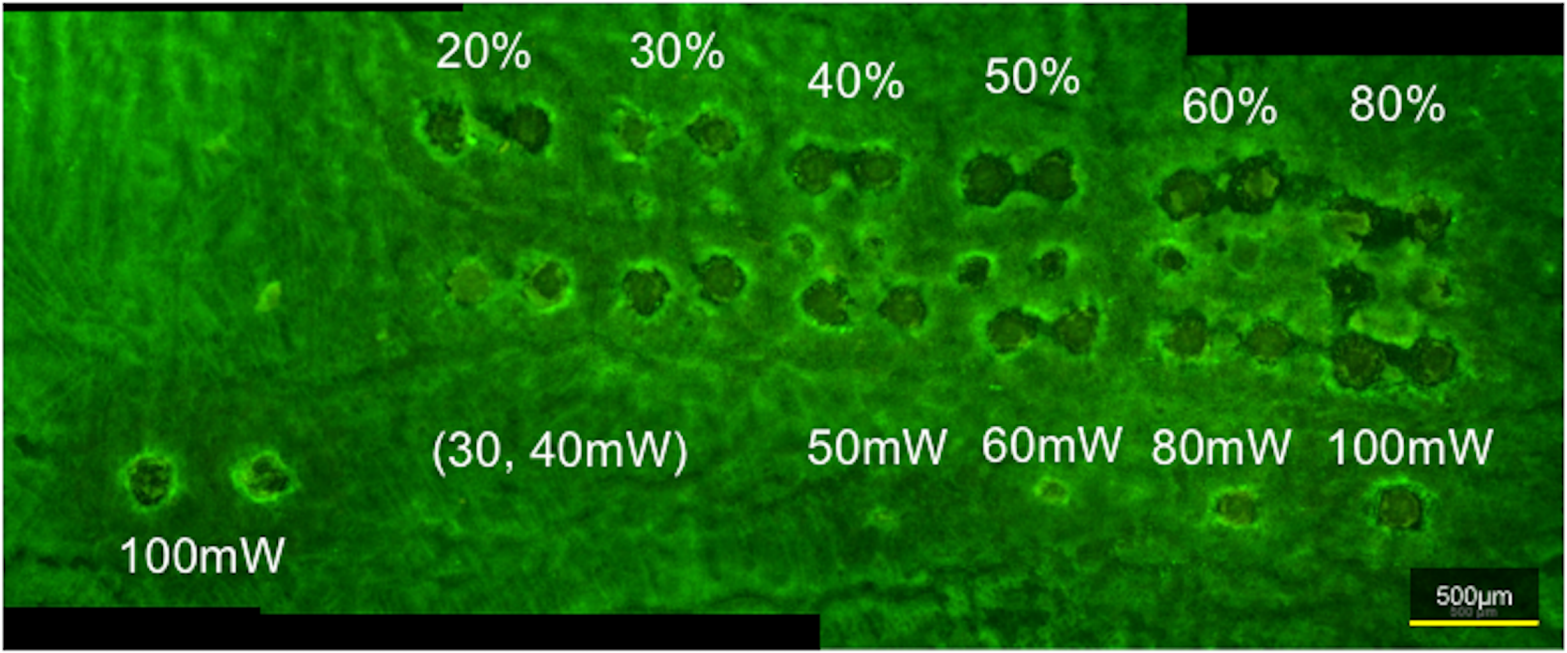
Panoramic photograph of fluorescent images of each laser spot. Panoramic photograph of fluorescent images obtained using confocal laser microscopy (original magnification ×60) after immunostaining of extension specimens with mouse anti-rhodopsin, and showing each laser spot. The percentages and mW indicates the energy setting of each method.

**Fig 4:**
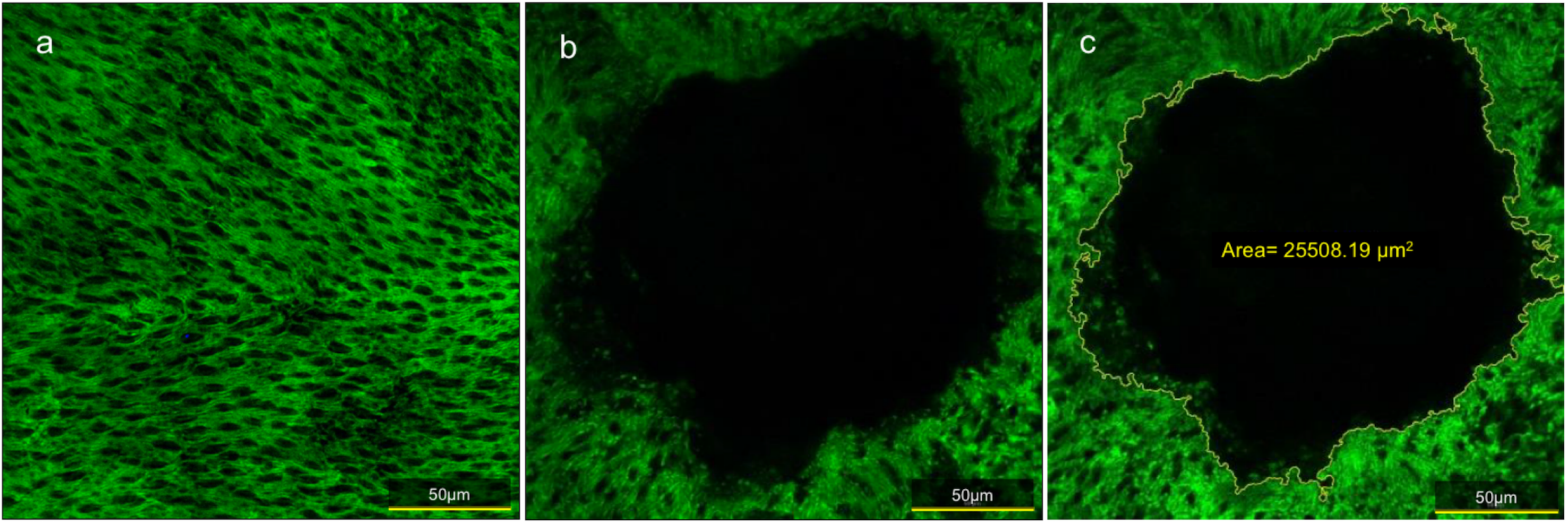
Method for measuring laser spots. (a) Enlarged photograph of anti-rhodopsin staining at the normal site of retina. (b) Spot created by laser application. (c) The dropout area of anti-rhodopsin staining was measured and calculated as the area of the laser spot.

The specimens were observed under a laser confocal microscope (C2, Nikon, Tokyo, Japan). The rhodopsin-stained retinal tissue was used to compare the areas of laser spotting using each method (Fig 3). The dropout area of anti-rhodopsin staining was measured and calculated, and defined as the area of the laser spot (the auto detect function implemented in NIS-Elements AR Analysis [Nikon] was used.) (Fig 4).

### 2.5. Coagulation spot comparison, OCT and histopathological findings at each energy in the three groups

**Fig 5:**
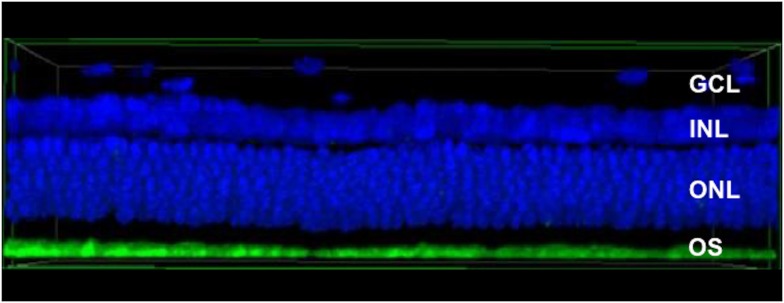
Construction of three-dimensional histopathological images to identify and compare the extent of tissue damage. Histological findings were used to re-construct three-dimensional images in side view with a center thickness of 15 μm using a confocal laser scanning fluorescence microscope system. The blue-color stained by the photoreceptor nucleus using DAPI correspond with the ganglion cell layer (GCL), the inner nuclear layer (INL), and the outer nuclear layer (ONL). The green-color stained by mouse anti-rhodopsin corresponds to the outer segment. This image is a normal structure, with no distortion or tissue dropout.

Ophthalmological findings from laser spots created by gradually decreasing the laser energies were compared among the three groups. To identify and compare the extent of tissue damage, OCT and histological findings were used to construct a three-dimensional histopathology image in side view at a center thickness of 15 μm using a confocal laser scanning fluorescence microscope (Fig 5). Based on OCT findings, the extent of tissue damage at the spot lesion was identified to the outer plexiform layer (OPL) or more inner region and defined as full thickness (FT). For other evaluations, the inner nuclear layer (INL), outer nuclear layer (ONL), or inner/outer segment junction (IS/OS) were defined.

Ganglion cell layers (GCLs), INL, and ONL were identified in the three-dimensional histopathology images, and the extent of tissue damage was evaluated. The image review was conducted by two retinal specialists. The area of the laser spot, and the extent of tissue damage according to OCT and histological findings, were compared among the three groups to estimate which energy levels to acquire when using the control method to obtain approximate laser spotting with EM.

### 2.5. Statistical analysis

Statistical analysis included one-way ANOVA to compare coagulation spot areas among the three groups; *P*<0.05 was considered to be statistically significant. Statistical analyses were performed using EZR (Saitama Medical Center, Jichi Medical University, Saitama, Japan), which is a graphical user interface for R (The R Foundation for Statistical Computing, Vienna, Austria). More specifically, it is a modified version of “R Commander,” designed to add statistical functions frequently used in biostatistics [11].

## Results

The mean area of spot size in each group and the number of spots that could be measured are summarized in Table 1. The area of the spots decreased as the energy gradually decreased in all groups. Because the PSS-SDM and VX-SDM systems could not deliver energy parameters of 20 mW to 10 mW, these were not evaluated. There were no differences that could be statistically compared area of spot size between 100% of EM, 100 mW of PSS-SDM and 100 mW of VX-SDM (P = 0.111 [one-way ANOVA]).

**Table 1:** Comparison of area of irradiation spots among the three treatment methods.

EM			PSS-SDM			VX-SDM	
Energy,%	Spot area (μm^2^)	Spots, n/n	Energy, mW	Spot area (μm^2^)	Spots, n/n	Spot area (μm^2^)	Spots, n/n
100	23,550±2453	7/7	100	20,218±962	5/5	15,713±1075	4/4
80	17,353±2598	8/8	80	16,253±1642	3/3	13,430±1145	3/3
60	10,268±1233	8/8	60	10,279±2557	3/3	9479±1585	3/3
50	7183±1082	8/8	50	5503±2445	2/3	7423±1900	3/3
40	4990±1394	8/8	40	N/A	0/3	6146±2779	3/3
30	4108±1918	5/8	30	N/A	0/3	5941±3642	2/3
20	2697±882	8/8	20	➖	➖	➖	➖
10	N/A	0/8	10	➖	➖	➖	➖

Data presented as mean ± standard error.

n/n = the number of spots that could be analyzed among the laser treatments. EM = Endpoint Management, PSS-SDM = PASCAL short-duration method (Streamline 577™, Topcon Medical Laser Systems, Inc., Santa Clara, CA, USA), N/A = not available; VX-SDM = Vixi short duration method (MC 500-Vixi™, NIDEK, Gamagori, Japan)

**Fig 6:**
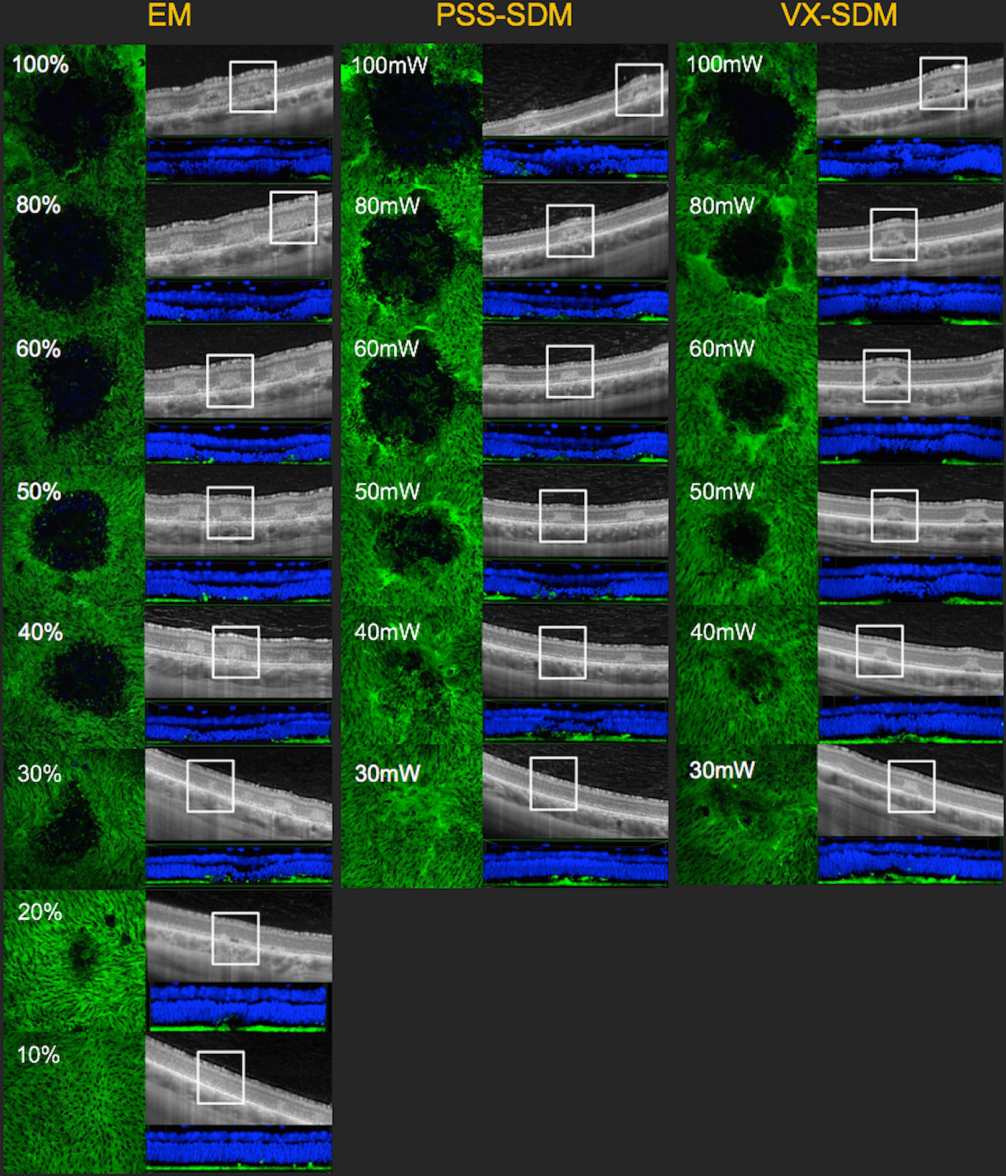
Comparison of grades in three different types of laser methods. Using Endpoint Management (EM), the laser spots were visible at 100% to 20%, and invisible at 10%. The tissue effects were identifiable at 20%; however, tissue damage was not apparent in 10% and appeared as normal structure. The extent of tissue damage in the outer nuclear layer at 30%, in inner segment/outer segment at 20% were detected by optical coherence tomography (OCT), and structural irregularity in outer nuclear layer (ONL) was identified at 20%, whereas 10% had normal structure according to three-dimensional histopathological imaging.

Table 2 and Fig 6 summarize the ophthalmologic findings of laser spots, the evaluation of damaged lesions according to OCT findings, and three-dimensional histopathological images according to gradual reductions in laser energy in each group. According to the ophthalmologic findings, the laser spots were visible up to 20%, 40 mW, and 30 mW with EM, PSS-SDM and VX-SDM, respectively (PSS-SDM and VX-SDM could not provide parameter settings below 20 mW).

**Table 2:** Extent of retinal tissue damage according to histopathological findings.

Visualization		Energy (%, mW)							
		100	80	60	50	40	30	20	10
Ophthalmologic findings	EM	+	+	+	+	+	+	+	N/A
	PSS-SDM	+	+	+	+	N/A	N/A	−	−
	VX-SDM	+	+	+	+	+	+	−	−
OCT findings	EM	FT	FT	FT	ONL	ONL	ONL	IS/OS	N/A
	PSS-SDM	FT	FT	ONL	ONL	N/A	N/A	−	−
	VX-SDM	FT	FT	ONL	ONL	ONL	ONL	−	−
Three-dimensional histopathology	EM	INL	INL	INL	INL	INL	INL	ONL	N/A
	PSS-SDM	INL	INL	INL	INL	INL	N/A	−	−
	VX-SDM	INL	INL	INL	INL	ONL	ONL	−	−

EM = Endpoint Management, FT = full thickness, ONL = outer nuclear layer, OPL = outer plexiform layer, OCT = Optical coherence tomography, IS/OS = inner/outer segment junction, INL = inner nuclear layer, PSS-SDM = PASCAL short-duration method (Streamline 577™, Topcon Medical Laser Systems, Inc., Santa Clara, CA, USA), VX-SDM = Vixi short duration method (MC 500-Vixi™, NIDEK, Gamagori, Japan)

Regarding the evaluation by OCT and three-dimensional histopathological imaging using EM, tissue effects were identifiable at an energy level of 20% using this method; however, tissue damage was not apparent at 10%, and appeared as normal structures. In PSS-SDM, tissue structure was normal at 40 mW according to OCT and 30 mW according to three-dimensional histopathological imaging. In VX-SDM, laser spots were detected up to 30 mW of the minimum energy setting that could be delivered, and structural irregularity of retinal tissue was observed at the same energy in the three-dimensional histopathological images.

Laser spots using EM were identifiable under the lowest power (20%) among the three groups. In contrast, 40 mW to 30 mW in PSS-SDM did not affect retinal structure; consequently, it was the method in which laser spots were most difficult to create. Regarding the comparison of spot size and tissue damage according to energy parameter in each group: the spot size at 50% setting on EM was 7183 μm^2^; PSS-SDM required 50 mW (5503 μm^2^) to 60 mW (10279 μm^2^) and VX-SDM required 50 mW (7423 μm^2^) to create the approximate spot size.

Furthermore, under the 50% setting on EM, 50 mW of PSS-SDM and VX-SDM, the extent of tissue damage in all three methods were generally in accord with ONL by OCT and INL by three-dimensional histopathological imaging. This result revealed that laser spots equivalent to 50% setting on EM can be obtained from PSS-SDM at 50 mW to 60 mW, and VX-SDM at 50 mW. However, for the 30% setting on EM, although the extent of tissue damage to the ONL was in accordance with OCT and INL with three-dimensional histopathological imaging at 50 mW in PSS-SDM and 50 mW in VX-SDM, similar laser spots could not be created from the perspective of laser spot area because the areas were 5503 μm^2^ at 50 mW in PSS-SDM and 7423 μm^2^ at 50 mW in VX-SDM, which was larger than the 4108 μm^2^ using the EM setting.

In the PASCAL (Streamline 577™, Topcon Medical Laser Systems, Inc., Santa Clara, CA, USA) short-duration method (PSS-SDM), the margin of laser spot became unclear at less than 40 mW. The spot identified by OCT in ONL at 50 mW, and at 30 mW was almost normal in structure in OCT and three-dimensional histopathological imaging.

In the Vixi (MC 500-Vixi™, NIDEK, Gamagori, Japan) short-duration method (VX-SDM), laser spots were identified using ophthalmologic visualization and OCT up to 30 mW; tissue damage was detected in the ONL. In the three-dimensional histopathological images, irregularities and distortions of the ONL were observed at 40 mW and 30 mW.

## Discussion

In this study, we investigated the possibility of laser application with EM using a conventional photocoagulator. Laser spots created by EM and control methods were assessed according to OCT and histological findings. Although the standard energy settings for EM are not well understood, generally, a threshold setting of 30% to 50% is often adopted. In our study, if the EM threshold setting of 50% is considered to be reasonable, the approximate spots were considered to be equivalent at 50 mW to 60 mW using PSS-SDM, or 50 mW using the VX-SDM. Although the extent of tissue damage at the 30% setting on EM corresponded to 50 mW in both the PSS-SDM and VX-SDM systems histologically, the spot sizes became larger than in EM using conventional models; therefore, we concluded that it was difficult to create an approximate laser spot using conventional models.

According to retinal tissue invasiveness, the predicted energy levels causing damage to the photoreceptor layer were 10% to 20% with EM, 40 mW to 50 mW with PSS-SDM, and 20 mW to 30 mW with VX-SDM. As a result, spot lesions using EM could be created using the least energy. This suggested that EM can be performed less invasively and more efficiently than conventional methods.

In a previous report investigating the retinal invasiveness of EM in rabbits, in which the laser settings were similar to ours (spot size 200 μm, duration 20 ms), tissue effects at 50% to 75% energy were not identifiable using ophthalmoscopic visualization methods, and OCT revealed visible spots at 100% to 200% in OPL, 50% to 75% in ONL, and did not reveal tissue damage at 30% [10]. Unlike that report, however, laser spots were detected at up to 20% using ophthalmoscopic visualization in the present study. We speculate that the outer segment (OS) was stained with mouse anti-rhodopsin, but its staining dropout by laser application was measured as a spot; therefore, the spots in our study could be detected at an even lower energy.

In evaluating the extent of tissue damage according to OCT, spot lesions were identifiable at FT at 60%, in the ONL at 30%, and in the IS/OS at 20%. Similar to ophthalmoscopic visualization, laser spots were also detected by OCT at lower energies in this study than in previous investigations. It is difficult to draw definitive conclusions about this outcome; however, we evaluated OCT findings immediately after laser treatment, while previous reports described results 1 h after laser treatment. Although our study used similar rabbits, it is also very likely that individual variation or limitations in the reproducibility of different photocoagulators may have been the reason for this this different result.

Although this study was based on the assumption of less invasive laser treatment using STLT in rabbits, previous studies have reported that histological analysis of patient lesions may reveal more extensive damage than rabbit histology of a given lesion intensity [12]. Therefore, in actual clinical practice, it is important to perform titration properly so as not to cause tissue damage due to intense burns. Moreover, racial and individual differences in clinical practice may be factors influencing the invasiveness of laser treatment for retinal tissue and, therefore, further studies are necessary. In recent years, retinal photocoagulator and photocoagulation methods have advanced: lighter lesions, which confine tissue damage in the photoreceptors and RPE layer, have become more common, [13] and a short pulse (10 ms to 30 ms) is commonly used in pattern scanning. The advantage of this method is the healing response to tissue damage compared with conventional methods [6]. When the outer layer of the retina is selectively coagulated using a short-pulse, there is a possibility that surrounding photoreceptor cells migrate to the damaged site over time and are repaired [14]; it has also been reported that laser spots disappear [10]. This is especially relevant for STLT using EM, which has a unique invasiveness adjustment in addition to a characteristically short pulse (0.015 s).

Consideration of retinal invasiveness is necessary when retinal photocoagulation is performed. However, setting the exposure time or energy to 50%, in an effort to simply reduce tissue invasiveness by 50%, does not necessarily mean it will actually be 50% (i.e., the relationship is not linear), despite the energy level being 50% mathematically. EM was designed by mapping computational model-based values of tissue damage (Arrhenius integral) to independently quantify tissue invasiveness and estimate the coagulation condition to set for the suitable treatment desired by the operators.

A previous study describing the therapeutic effects of STLT demonstrated that subthreshold micropulse diode laser photocoagulation for DME reduction was clinically effective [15], and had the similar effect of a modified ETDRS photocoagulation protocol [16], or was more effective [10], which is expected for the treatment of DME. However, the main challenge of STLT is to configure less invasive treatment by effective titration of settings. Although tissue damage is confined to a single RPE cell with a 30% setting on EM [10], this may be considered to be a treatment risk of STLT; consequently, an EM setting of 30% to 50% is recommended.

The treatment strategy for DME has also undergone a major change. In recent years, intravitreal injection of pharmacological anti-vascular endothelial growth factor (VEGF) agents has been used to effectively treat DME [17]; however, various problems remain. This anti-VEGF treatment is also associated with a risk for ocular infection, a very high cost to the healthcare system, and the burden of frequent intravitreal injections for patients and physicians.

Although laser treatment for retinal disease has, to date, been unable to yield an acceptable level of retinal invasiveness, the concept of retinal photocoagulation has been changing due to the development of new devices including the pattern scan laser, STLT such as EM, or micro-pulse technology. In addition, this study demonstrated that EM-approximated STLT could be performed with conventional photocoagulation without using specialized models for STLT. Although these results need further examination in clinical trials to extend the indications for STLT, which is less invasive compared with classic coagulation and has a longer-lasting effect than anti-VEGF therapy, it can contribute to improvement of the DME reduction effect, and may decrease the need for frequent administration of anti-VEGF performed alone or in combination with anti-VEGF administration, and thus, more effective treatment can be expected.
